# Editorial: TRP Channels in Inflammation and Immunity

**DOI:** 10.3389/fimmu.2021.684172

**Published:** 2021-04-15

**Authors:** Santiago Partida-Sanchez, Bimal N. Desai, Albrecht Schwab, Susanna Zierler

**Affiliations:** ^1^ Center for Microbial Pathogenesis, Nationwide Children’s Hospital & Department of Pediatrics, The Ohio State University, Columbus, OH, United States; ^2^ Department of Pharmacology, University of Virginia, Charlottesville, VA, United States; ^3^ Institute of Physiology II, University of Münster, Münster, Germany; ^4^ Walther Straub Institute of Pharmacology and Toxicology, Ludwig-Maximilians-Universität München, Munich, Germany; ^5^ Institute of Pharmacology, Johannes Kepler University Linz, Linz, Austria

**Keywords:** ion channels, inflammation, innate immunity, phagocytes, mast cells, NK cells

TRP channels respond to a variety of chemical and physical stimuli derived from harmful agents and contribute to increased intracellular cation concentrations. TRP channel-mediated effects in immune cells are diverse and range from regulation of cell migration and phagocytosis, to the production and release of inflammatory mediators. Furthermore, these channels mediate an active crosstalk between epithelial cells, neuronal tissue, and the immune cells that orchestrate immune responses to tissue damage or infection. The articles published in this special issue review current thinking with regards to the role of TRP channels in innate immune cells, covering from the prototypical phagocytic cells, neutrophils, and macrophages, to other innate immune cells like mast cells and natural killer cells. Original articles and reviews highlight the involvement of TRP channels in regulating the inflammatory properties of innate immune cells, and how these processes shape the outcome of diseases including inflammatory bowel disease, lung disease, allergic disorders, infectious and autoimmune diseases. Because of the wide functional role of TRP channels in inflammation and immunity, authors discuss the use of specific agonists or antagonists and the potential for development of novel treatments for infectious and/or inflammatory diseases.

In the special issue, several original papers focus on TRP channels in neutrophils. The work by Robledo-Avila et al. adds new knowledge to the role of TRPM2 channels in neutrophils during a L. monocytogenes infection. They show that a role of TRPM2 channels is to contain the inflammatory response to a L. monocytogenes infection. Accordingly, TRPM2^−/−^ mice develop septic shock and TRPM2^−/−^ neutrophils acquire a hyperinflammatory profile. The increased inflammatory properties of TRPM2^−/−^ neutrophils correlate with dysregulated cytoplasmic concentration of Ca^2+^, which potentiate membrane depolarization and accounts for the higher susceptibility of TRPM2^−/−^ mice to infection. Whereas the importance of TRPM2 in regulating the intracellular Ca^2+^ concentration ([Ca^2+^]i) in neutrophil function has been studied, TRPM2 as non-selective cation channel, is also permeable to Na^+^. However, the impact of this channel in modulating the intracellular Na^+^ concentration ([Na^+^]_i_) is unknown. The original research article by Najder et al. exams for the first time how knockdown of TRPM2 may affect the intracellular Na^+^ homeostasis and chemotaxis of neutrophils. The authors found that knockout of TRPM2 channel results in altered neutrophil [Na^+^]_i_, likely by indirectly modulating the Na^+^ transport protein NCX1. Therefore, they postulated that TRPM2 channel regulation of cation balances may be essential under inflammatory environment.

TRPM2 channel is widely expressed in immune cells and it is firmly established that the channel can be activated by intracellular adenosine 5′-diphosphoribose (ADPR). However, additional NAD derivatives including NAADP and cADPR have been considered TRPM2 agonists, but the activating mechanism and activation site of the channel for these nucleotides remain controversial. In a review article Fliegert et al. critically summarize the literature regarding the role for cADPR as an agonist of TRPM2 channel, with an emphasis on recent structural data. Authors conclude from the literature that removal of contaminating ADPR prevents activation of TRPM2 by commercial cADPR, nonetheless, the idea that cADPR could affect TRPM2 channel remains unsettled.

Another original research article by Nadolni et al. investigates the role of TRPM7 channels in neutrophil inflammatory recruitment with a particular focus on the kinase domain of this channel. They found that mice whose TRPM7 channels bear an inactive kinase domain are recruited less efficiently in a peritonitis model. This is underpinned by *in vitro* studies that reveal reduced chemotaxis and reactive oxygen species production in the presence of TRPM7 channel or kinase blockers.

TRPV4, members of the vanilloid (TRPV) subfamily of TRP channels, are widely expressed mechanosensitive channels also found in many (innate) immune cells, where they contribute to their activation and differentiation. Immune cells are challenged by many mechanical cues that may originate from the blood stream during the recruitment process, from the surrounding extracellular matrix or be a consequence of the respective organ function such as ventilation or heartbeat. The new concept of mechanoimmunology attempts to bring the function of immune cells and their response to mechanical cues in a causal relationship. Two reviews are evaluating this idea with TRPV4 channels being the common denominator. Michalick and Kuebler provide an overview on how the mechano-sensitivity of TRPV4 channels (“mechanoTRPV4”) impacts on their role in immune cells (“immunoTRPV4”). Scheraga et al. discus the link between mechanics and signaling in a narrower context for macrophages and lung injury. Furthermore, Alpizar et al. report in an original research that TRPV4 mediates LPS-induction of inflammatory innate immune responses in urothelial cells. TRPV4 is highly expressed in urothelial cells and is known to play a role in sensing the normal filling state of the bladder and the mechanisms of bladder voiding. The regulatory role of TRPV4 on the increase in proinflammatory cytokine expression induced by bacterial LPS is a novel finding.

Mast cells and basophils are essential drivers of allergic and anaphylactic reactions. Two original papers focus on the role of TRPC channels in these innate immune cell types. Tsvilovskyy et al. nicely demonstrate that TRPC channels contribute at least partially to mast cell activation *via* the Mas-related G-protein coupled receptor member B2 (MrgprB2), a mast cell-specific receptor for basic secretagogues, such as the widely used compound 48/80 or substance P. On the other hand, mast cell activation following FcϵRI stimulation is not affected by TRPC channels. Authors support their claim that TPRC channels might be associated with systemic pseudo-allergic reactions by using double (Trpc1/4^−/−)^ and triple (Trpc1/4/6^−/−^) knockout mouse models and a thorough comparison of bone marrow derived mast cells versus peritoneal mast cells. In the other study, Bacsa et al. elegantly test the principal possibility of pharmaco-optogenetic modulation of the function of immune cells using a recently established TRPC3/6/7 selective photochromic benzimidazole agonist OptoBI-1. The researchers demonstrated that the rat basophilic cell line, RBL-2H3, lacks noticeable Ca^2+^/NFAT signaling in response to OptoBI-1 photocycling. Different genetic modifications of these cells, by introduction of recombinant benzimidazole-sensitive TRPC isoforms (TRPC3/6/7), revealed that exclusively the single expression of TRPC6 generates OptoBI sensitivity suitable for opto-chemical control of NFAT1 activity. These results provide the first proof-of-concept for efficient chemo-genetic targeting of Ca^2+^ signaling in basophils and transcriptional regulation based on TRPC photo-pharmacology.

Unlike most TRP channels, which are expressed at the plasma membrane, endolysosomal TRP mucolipin channel family (TRPML), are found in the endolysosomal system. Endosomes and lysosomes are involved in many aspects of immune cell function, such as phagocytosis, antigen presentation and processing by antigen-presenting cells, release of proinflammatory mediators. Consequently, TRPML channels must play critical roles in inflammation and immunity. In this special issue, Spix et al. critically review the multiple functions of TRPML channels in regulating immune responses. This includes the TRPML1-mediated modulation of secretory lysosomes, granzyme B content, tuning of effector function in NK cells, TRPML1-dependent directional dendritic cell (DC) migration and DC chemotaxis, and the role of TRPML2 in chemokine release from LPS-stimulated macrophages. However, they noted that expression and the functional roles for TRPML channels on other very diverse immune cell types (e.g. basophils, eosinophils, monocytes and T cells) remain to be elucidated. With a more specific focus on TRPML channels on NK cell functionality and cancer immunotherapy, Clement et al. provide a concise review of lysosome biogenesis in NK cells. They further highlight methodological advances and new specific pharmacological modulators generated in the last years and their potential for future studies.

In addition, Santoni et al. review the impact of TRPML channels on mammalian anti-viral responses as well as on NK cell function. They highlight, on the one hand, an essential role of TRPML1 and 2 channels in the promotion of virus entry and infectivity and, on the other hand, the contribution of TRPML channels enhancing antiviral innate and possibly adaptive immune responses by regulating TLR signaling in different innate immune cells. Thus, TRPML channels might form a double-edged sword in the innate immune response to viral infections.

There is an emerging theme that TRP channels play a diverse yet poorly defined role in the immune– nonimmune cell crosstalk during inflammatory processes. Chen et al. review and summarize the plethora of findings linking the functions of TRP channels in immune cells as well as sensory nerves in the pathogenesis of inflammatory bowel disease. Similarly, the review by Silverman et al. elaborates further on how neuronal TRP channels drive the inflammatory processes in general. These reviews clearly highlight the significance of TRP channels in inflammatory processes, but they also reveal that there are glaring mechanistic gaps in our understanding of how TRP channels and their downstream electrical signals regulate these inflammatory processes – much work is needed to understand how TRP channels regulate intracellular signaling in immune cells and how their activities help coordinate the complex cellular networks that underly tissue inflammation.

In summary, this special issue of Frontiers in Immunology presents a comprehensive overview of the most recent data on the role of several TRP channel families in innate immunity. It highlights the importance of these proteins as potential drug targets for future therapeutics against allergic reactions, inflammatory or infectious diseases.

## Author Contributions

All authors listed made direct intellectual contribution to the work, wrote, edited, and approved the final manuscript for publication.

## Funding

SP-S was supported by the Cystic Fibrosis Foundation grant PARTID18P0. BD was supported by NIH grants GM108989 and HL120840. AS was supported by Deutsche Forschungsgemeinschaft SCHW407/17-1 and IZKF Münster Schw2/020/18, and SZ was supported by the Deutsche Forschungsgemeinschaft (DFG) TRR-152/2 (P14).

## Conflict of Interest

The authors declare that the research was conducted in the absence of any commercial or financial relationships that could be construed as a potential conflict of interest.

